# Clinical profile and mortality among adult patients presenting with altered mental status to the emergency departments of a tertiary hospital in Tanzania: a descriptive cohort study

**DOI:** 10.11604/pamj.2022.41.46.31601

**Published:** 2022-01-18

**Authors:** Hussein Karim Manji, Hendry Robert Sawe, Said Kilindimo, Elishah Nuralah Premji, Amne Omar Yussuf, Alphonce Nsabi Simbila, Sabira Akber Versi, Ellen Joyce Weber

**Affiliations:** 1 Emergency Medicine Department, Muhimbili University of Health and Allied Science, Dar es Salaam, Tanzania,; 2 The Aga Khan Hospital, Dar es Salaam, Tanzania,; 3 Emergency Medicine Department, Muhimbili National Hospital, Dar es Salaam, Tanzania,; 4 Department of Emergency Medicine, University of California, San Francisco, California, United States of America

**Keywords:** Altered mental status, emergency department, Tanzania, sub-Saharan Africa

## Abstract

**Introduction:**

Altered mental status (AMS) in the Emergency Department (ED) can be associated with morbidity and mortality. In high income countries, mortality rate is under 10% for patients presenting with AMS. There is a paucity of data on the profile and mortality amongst this group of patients in limited income countries.

**Methods:**

this was a prospective cohort study of adults ≥18 years presenting to the Emergency Departments of Muhimbili National Hospital (MNH) Upanga and Mloganzila in Tanzania with Altered Mental Status (AMS) unrelated to psychiatric illness or trauma, from August 2019 to February 2020. Patient demographic data, clinical profile, disposition and 7-day outcome were recorded. The outcome of mortality was summarized using descriptive statistics.

**Results:**

among 26,125 patients presenting during the study period, 2,311 (8.9%) patients had AMS and after exclusion for trauma and psychiatric etiology, 226 (9.8%) patients were included. The median age was 56 years (43-69 years) and 127 (56.2%) were male. Confusion 88 (38.9%) was the most common presenting symptom. Hypertension 121 (53.5%) was the most frequent associated comorbidity. The overall mortality was 80 (35.4%) within 7 days. Of 173 patients admitted to the wards, 54 (31.2%) died and of the 46 (20.4%) admitted to the intensive care unit (ICU), 20 (43.5%) died within 7 days. Six (2.7%) patients died in the emergency department.

**Conclusion:**

patients with AMS presenting to two EDs in Tanzania have substantially higher mortality than reported from Hospital Incident Command System (HICS). This could be due to underlying disease, comorbidities or management. Further research could help identify individual etiologies involved and high risk groups which can cater to better understanding this population.

## Introduction

Altered mental status is a common yet highly non-specific presentation of patients to the Emergency Medicine Department (EMD) [1]. Altered mental status (AMS) is a broad general term which is employed to describe a constellation of disorders associated with mental dysfunction ranging from slight confusion to coma. AMS includes a variety of specific conditions including delirium, stupor, coma and dementia [2]. The term is said to be vague and has many synonyms often referred to by non-medical personnel or the layman as confusion, not acting right, altered behavior, altered sensorium, generalized weakness, lethargy, agitation, psychosis, disorientation, inappropriate behavior, inattention, and hallucination [3,4]. Acute presentation of altered mental status may be the presentation of a patient as a result of underlying disease conditions including structural, metabolic, vascular or other conditions [5-7] or may be a late manifestation due to hypoxia or hypoperfusion to the brain [8]. It may also be the first symptom of a more morbid condition like a cerebrovascular event or an element of a diagnosis itself like dementia [2,9].

Studies in high and upper middle-income countries have found patients presenting with AMS have a bimodal distribution of ages with mortality rates as high as 10% [10,11]. Studies done in high income countries also demonstrate altered mental status significant increases the risk of mortality in many conditions. Altered mental status was a significant predictor of death in patients with community acquired pneumonia [5], hypothermia [7], cardiogenic shock [6], and most recently COVID-19 [8]. One study found that patients presenting with AMS have worse short-term prognosis and mortality rates than patients presenting to the ED with chest pain [12].

In studies in Africa the mortality associated with AMS appears to be much higher. Most patients with AMS belong to a younger age group and a significant number were HIV positive [13-15]. There is a paucity of studies conducted in relation to this presentation in low-income settings and therefore very scarce data on the profile and outcomes of such patients. It is not clear if the higher mortality seen in our setting is due to a higher severity on presentation, the etiology of the condition or lack of appropriate care.

In Tanzania, as in much of sub-Saharan Africa, the infrastructure for emergency care is still in development. Little is known about the number and clinical profile of patients with AMS presenting to the EMD, or their outcome. This study aimed to describe the clinical profiles disposition and mortality amongst adult patients presenting with altered mental status to the emergency department.

## Methods

**Study design:** this was a prospective cohort study of adult patients aged 18 years and above presenting to the EMD-MNH Upanga and Mloganzila with altered mental status between August 2019 and February 2020.

**Study setting:** this was a two-centered study, conducted in the Emergency Departments of Muhimbili National Hospital (MNH) and MNH Mloganzila (MAMC) in Dar es Salaam, Tanzania. MNH Upanga, located in the Ilala District, serves as the National Referral Hospital and University Teaching Hospital with 1,500 bed facility, attending 1,000 to 1,200 outpatients per week, and admitting 1,000 to 1,200 inpatients per week. The Emergency Medicine Department (EMD) receives all emergency referrals and cases at Muhimbili National Hospital and provides services for acutely ill patients with life threatening conditions. The department was established in January 2010 via a partnership between the Ministry of Health and Social Welfare and the Abbott Fund Tanzania. The EMD is the first and currently only full capacity emergency medicine department in Tanzania having emergency physicians available in the department for 24-hours every day including weekends. The EMD attends to critically ill patients at an average of around 200 per day, conducts research in emergency medicine, and trains emergency care providers for the country, hosting the only emergency medicine residency program in Tanzania. The MNH ED MNH also has point of care tests readily available to evaluate renal function, electrolytes and other potential causes of altered mental status. MNH Mloganzila (MAMC) is a national specialty and super specialty hospital, and a premier center of teaching health care professionals, services provision and research in Tanzania. It is a 1,500 bed capacity facility, attending 1,000 to 1,200 outpatients per week, and admitting 1,000 to 1,200 inpatients per week, It was officially inaugurated on November 25^th^, 2017. MAMC is a government owned hospital which serves patients from across all regions of United Republic of Tanzania who are referred from regional referral and designated hospitals for medical intervention. It is equipped with a state-of-the-art emergency medicine department, occupying 3,800 acres and is located 3 kms off the Dar Es Salaam (DSM)-Morogoro highway in Mloganzila.

The EMDs in both the centers are staffed with highly trained teams of medical personnel with 24 hours specialist coverage. Both facilities have specialists in neurosurgery, neurology, infectious disease, endocrinology and psychiatry on call on a daily basis. Both facilities also have availability of Computed Tomography (CT) scans as well as Magnetic Resonance Imaging (MRI) and electroencephalogram (EEG). To aid in the on-going management of the critically ill altered patient, a well-staffed and equipped ICU with a capacity of around 30 beds is available in both centers as well as respective wards for disposition after initial stabilization.

**Study participants:** all adults (age greater than or equal to 18 years) presenting with AMS unrelated to trauma or a known psychiatric illness were eligible for the study. We excluded patients who had previously been enrolled in the study who presented with recurrent episodes of AMS during the period of the study. The exclusion criteria were identified based on patient delineation and triaging in the respective departments where known psychiatric patients with acute symptoms are triaged and managed separately and also to identify causes and comorbidities related to altered mental status due to non-psychiatric and non-trauma related causes which may not be as straightforward as the two. All patients were entered into the study after an informed consent was signed by relatives/guardians. The investigator received a waiver of consent from the University Institutional Review Board in case of the absence of relatives to provide consent.

**Variables: the variables of interest were**

**Independent variables:** a) Demographic data: age, sex, marital status and occupation; b) referral status: self-referred/hospital referred; c) insurance status: insured/cash paying or public; d) comorbidities: prior H/o similar episodes, renal disease, liver disease, diabetes mellitus, hypertension, HIV/AIDS, epilepsy, malignancy, trauma, known psychiatric illness; e) drug use within 24-48 hours: alcohol, illicit drug use, anticonvulsants, sedatives, prescription pills (for possible interactions); f) prior H/o hospitalization due to AMS; g) clinical severity: clinical - Glasgow Coma Scale (GCS); h) vital signs at presentation and on disposition; i) associated symptoms: fever, difficulty breathing, pain, focal neurological deficits, excessive sweating / diaphoresis, vomiting; j) ED management strategies: fluid resuscitation, supplemental oxygen, bedside glucose testing, cardiac monitoring, sedation, antidote administration, antibiotics, septic workup, external service consultation, brain imaging (CT/MRI/EEG); k) disposition status.

**Outcome variables:** mortality: ED mortality and 7 days.

**Data sources/measurement:** a consecutive patient enrollment scheme was applied. The principal researcher and his assistant(s) were available in the ED 24 hours a day, seven days a week to ensure all eligible patients were captured during the period of study. All the doctors and nurses at the beginning of the shift were advised to notify either the researcher or his assistants in case they received any patient with AMS or any patient with a GCS <15. Additionally, research assistant(s) monitored the electronic tracking board for chief complaints related to AMS (e.g. confusion, loss of consciousness, convulsions, etc.). Following the triaging process, patients with triage complaint of either confusion, altered mental status, convulsions, stupor, coma, delirium, abnormal behavior, loss of consciousness, disorientation or assigned a GCS less than 15 were approached to determine eligibility. A standardized data collection form was used to assess the eligibility of patients to enroll in the study. An opportunity to participate in the study for the patient was offered to patient relatives (proxy) by providing them an information sheet and providing them all the relevant information concerning the study and through the social worker as proxy for patients with no relatives.

Four research assistants were recruited to facilitate a continuous data collection process on both day and night shifts in both the study centers. Prior to data collection, the research assistant(s) were trained on identification of patients with AMS, the use of the data collection checklist, and the relevant information to be collected. All admitted patients were followed up in a hospital ward or and through mobile phone calls post discharge within 7 days to determine their outcome from the ED-MNH and MAMC, and 24 hour and 7-day outcome was recorded. The principal researcher oversaw the data collection process and data were verified for quality and accuracy on a daily basis. The data were collected under strict supervision for consistency and records were rechecked and verified daily.

**Study size:** sample size estimate was based on a planned secondary analysis of risk factors for mortality. Age is an important determinant of mortality in patients with AMS presenting to the EMD. A pilot study was done in the EMD from January to February 2019 which showed 60% of patients with AMS were more than 60 years of age and 40% were between 18 to 60 years of age. The overall mortality was 20% with 30% deaths within the >60 years age group and 14% in the group between 18-60 years. The sample size for this study was calculated using the following formula [16-18]:


$$N=\frac{z\alpha \sqrt{P(1-P)(1/q1+1/q2)}+z\beta \sqrt{P1(1-P1)(1/q1)}+P2(1-P2){\left(1/q2\right)}^{2}}{{\left(P1-P2\right)}^{2}}$$

Where α represents the group of patients with AMS >60 years of age; β represents the group of patients with AMS between 18 to 60 years; the standard normal deviate for α which is Zα = 1.960; the standard normal deviate for β which is Zβ = 0.842; P1 is the proportion of patients who died in the group >60 years; P2 is the proportion of patients who died in the group 18-60 years; q1 is 1 minus P1 which represents the proportion of those who survived in the first group; q2 is 1 minus P2 which represents the proportion of those who survived in the second group; total group size = N = (A+B)2/C = 200. The minimum sample size was 221 patients after adjustment was made for 10% loss to follow up rate.

**Statistical methods:** data were coded and imported into Research Electronic Data Capture (REDCap). Statistical Package for Social Science (IBM SPSS version 25, IBM, LTD, Carolina, USA) was used for analysis. Descriptive statistics were computed with continuous variables presented as median (IQR). Categorical variables including demographics, clinical presentations and disposition were expressed as number and percentage.

**Ethical consideration:** the study was conducted after obtaining permission from the University Institutional Review Board and National Hospital. All patients were entered into the study after an informed consent was signed by the relatives/ guardians. The investigator received the waiver of consent from the University Institutional Review Board in case of absence of relatives to provide consent. All patients received treatment as per standard hospital policies. The data obtained during the study was kept anonymous. The data were coded to hide patient´s identity and stored in computer with password known by researcher only. The written forms were kept in a safe locker accessible by the researcher only.

## Results

**Participants enrolled in the study:** of the total 26,125 adult EMD visits, we identified 2,311 (8.9%) patients with AMS. Two thousand and sixty-five patients were excluded due to a known psychiatric illness or trauma related presentation and 20 excluded as revisits. Of the 226 patients included in the study, all but one was admitted to the hospital with 46 (20.1%) admitted to ICU and 173 (76.5%) to the ward.

**Descriptive data:** median age was 56 and there was a higher proportion of males. The most commonly encountered presenting symptom (per the treating physician) was confusion followed by loss of consciousness and disorientation while the most common associated symptoms were focal neurological deficits and difficulty breathing. The most common comorbidity was hypertension, followed by diabetes (Table 1).

**Table 1:** demographics and clinical profile of adult patients with altered mental status presenting to the Emergency Departments of Muhimbili National Hospital, Dar es Salaam, Tanzania (N=226)

Variable	Frequency n (%)
**Sex**	
Male	127 (56.2)
**Age**	
18-60 years	127 (56.2)
Median age (IQR)	56 (43-69)
**Occupation**	
Employed	61 (27.0)
**Referral status**	
Hospital - referred	126 (55.8)
**Presenting symptoms**	
Confusion	88 (38.9)
Disorientation	50 (22.1)
Unconscious	57 (25.2)
**Comorbidities**	
Hypertension	121 (53.5)
Diabetes mellitus	51 (22.6)
Previous episode of AMS	33 (14.6)
HIV	14 (6.2)
**Associated symptoms**	
Focal neurological deficit	49 (21.7)
Difficulty breathing	46 (20.4)

IQR: interquartile range

**Figure 1:**
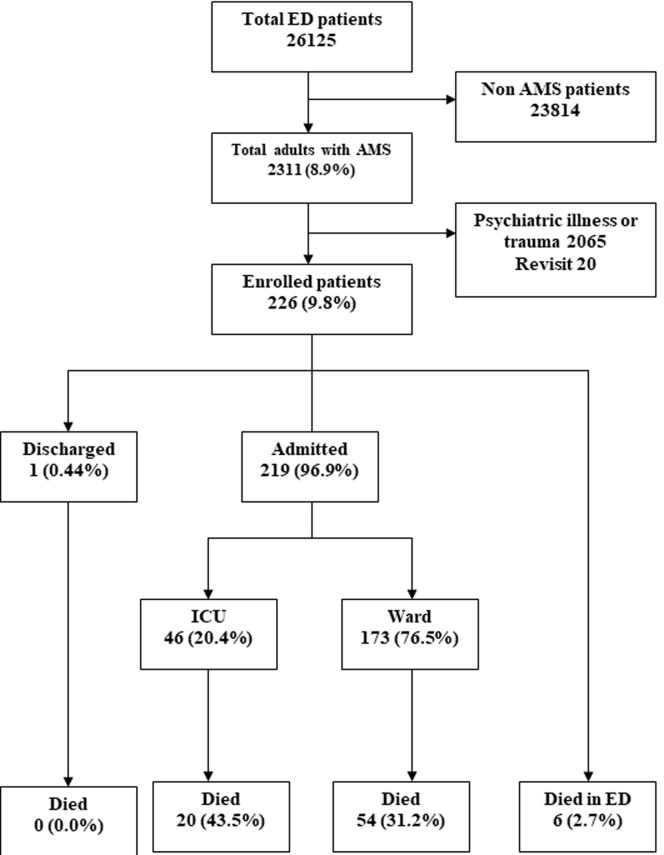
flow chart of adult patients presenting to the study departments with altered mental status during the study period

**Outcome data:** follow-up was achieved on all patients. Of the 173 patients admitted to the general wards, 151 (66.8%) were admitted under the internal medicine department amongst whom 76 (33.6%) were admitted under neurology followed by 23 (10.2%) under infectious disease (Table 2). Of those admitted to the ICU, 26 (56.5%) died within seven days. Of those admitted to wards, 74 (32.7%) died by the end of 7 days. Death in the EMD occurred in 2.7% percent of the total study population (Table 3). The overall mortality including the deaths in the EMD was 80 out of 226 enrolled patients (35.4%) (Figure 1).

**Table 2:** disposition amongst adult patients with altered mental status presenting to the Emergency Departments of Muhimbili National Hospital, Dar es Salaam, Tanzania (N=226)

Disposition	Total n (%)
Internal medicine	151 (66.8)
Neurology	76 (33.6)
Infectious disease	23 (10.2)
Gastroenterology	19 (8.4)
Nephrology	19 (8.4)
Endocrinology	11 (4.9)
Pulmonary medicine	2 (0.9)
Hematology	1 (0.4)
ICU	46 (20.4)
Neurosurgery	11 (4.9)
Surgery	5 (2.2)
Cardiology	3 (1.3)
OBS / GYN	2 (0.9)
Urology	1 (0.4)

ICU: intensive care unit; OBS: obstetrician; GYN: gynecologist

**Table 3:** mortality amongst adult patients with altered mental status presenting to the Emergency Departments of Muhimbili National Hospital, Dar es Salaam, Tanzania (N=226)

Disposition	Total n (%)	Died in the EMD	Died within 7 days n (%)	Lived n (%)
Discharged from ED	1 (0.4)	-	-	1 (100.0)
Admitted (ward)	173 (76.5)	-	54 (31.2)	119 (68.8)
Admitted to ICU	46 (20.4)	-	20 (43.5)	26 (56.5)
Total deaths	80 (35.4)	6 (2.7)	74 (32.7)	

EMD: emergency medicine department; ED: emergency department; ICU: intensive care unit

## Discussion

Altered mental status (AMS) is a diagnostic challenge in the EMD with a multitude of possible etiologies. Amongst the patients with AMS seen in these two EDs, the overall in-hospital mortality rate was 35.4% including the deaths in the EMD. This mortality rate corresponds to other studies done in Africa [13-15] but is significantly higher in comparison to studies done in high income countries [10,11,19,20]. The high mortality rate suggests both gaps in stabilization and care of patients presenting with AMS as well as multiple demographic and clinical factors which contribute to this high rate of death.

Care in the ICU is expected to lead to improved survival. The finding that mortality rate was higher in the ICU than the ward is likely explained by the clinical condition of patients, where only the most seriously ill are placed in the ICU in our setting. High mortality in the ICU is a finding seen in other studies [21]. However, more detailed studies are warranted to investigate any gaps in care in the intensive care units. Majority of the patients were admitted to internal medicine department with neurology and infectious disease being the leading sites for disposition. This indicates that besides neurological causes directly affecting brain function, AMS in many in our study was due to systemic illness where the brain suffers end organ damage. This finding is supported by studies, both within African countries and non-African countries where neurological and infectious etiologies were the dominant causes of AMS in patients [10,13-15,20].

Among all adult patients presenting to the EMD in this study, the proportion of patients with altered mental status was found to be 8.9%. There is no study in African countries which clearly demonstrated or highlighted the prevalence of AMS; but this finding correlated with some studies done outside Africa [11,22,23]. This indicates that AMS constitutes a huge burden in our setting and significantly plays a role in the influx of patients to the emergency department. It also further emphasizes the need for emergency physicians to be equipped in the approach and management of patients with AMS as these patients contribute a substantial amount to the overall ED burden. One frequently taught mnemonic for evaluating AMS is the using the AEIOU-TIPS approach [24].

The median age of our patients was 56, consistent with studies done in Asia and in other non-African countries [10,19] but different from studies done in other African countries, where patients with altered mental status were generally younger [13-15]. This finding may be related to the fact that our ED is a referral hospital; AMS in younger patients tends to be more transient due to drug use and may therefore they not be referred to referral hospitals. Elderly patients are also more likely to have multiple vulnerability factors hence being more susceptible to becoming delirious compared to their younger counterparts [4,22]. Most of our patients were male, a finding corresponding to studies done both in African and non-African countries [10,14].

Confusion was the most common chief complaint. This complaint was generally assigned by the triage nurse, often based on what a relative would have said. There are many possible terms as regards AMS, more than 25 as per one count [25], most triaging personnel may not be familiar with the broad definition of all terms in the altered patient and hence employ the term ‘confusion´ for most altered patients. Confusion is often based on the judgment of a relative or second person as behavior that is deemed unusual for the individual or deviates from societal norms; and patients do not complain of confusion, consistent with their lack of insight into their behavior [26].

Precisely defining AMS has been shown to be useful in diagnosing the underlying condition. It is important for the physician to use an appropriate assessment method to identify and quickly stratify patients presenting with confusion early and ensure the interventions required are implemented at the earliest. The Confusion Assessment Method (CAM) is one such screening test which can be employed to further improve the information available.

This symptom was closely followed by loss of consciousness and disorientation. This is in conjunction to some studies where impaired cognition, impaired consciousness or delirium were the major presentations [27]. Some studies have used terms synonymous including inattention, unresponsive, unusual behavior and impaired mental performance as the initial presentations [28,29]. The emphasis should be on educating ED physicians from triage onward on the definition of all key terms and their applicability and indication as this may serve as clues to the ultimate diagnosis of the patient as well.

The most common comorbidity was hypertension which can be associated with AMS by causing hypertensive encephalopathy, renal failure with uremia and stroke; however, hypertension is a very common condition worldwide and may not have had a direct relation to the cause of AMS [30]. The proportion of patients with HIV in our study was notably lower than in previous studies of AMS patients in Africa [13,15]. This may indicate improvements in surveillance systems and care of HIV positive patients as studies show that between 2010 and 2018, the number of new infections declined by 13% and the number of people dying from an AIDS-related illness has halved [31].

**Strengths:** this was a two centered prospective study with consecutive recruitment of participants. This aided in improving representativeness and reducing selection bias. Furthermore, all patients were followed up in this study. Records underwent quality assurance checks daily by the predictive index (PI) to avoid any flaws in data collection. Findings can be generalized as sample was representative of study population, simple methods of analysis were employed and the data was consistent, reliable and precise.

**Limitations:** patient profiles and comorbidities were assessed based on information from relatives or prior hospital documentation brought with the patient, which could have over or under stated some co-morbidity and affected the results. Management strategies, and disposition were entirely physician dependent and no active interventions were done; physician to physician variability in clinical decision making may have played a role in the overall results.

## Conclusion

This study highlights that outcome in patients with AMS are poor in Tanzania. Future studies should focus on risk factors for worse outcome including both clinical profiles and management.

### What is known about this topic

- *Altered mental status is a common symptom complex presenting to the emergency department;*
- *Multiple etiologies can cause a patient to develop an alteration in their mental status;*
- *Patients presenting with these symptoms have significant mortality risks as per other studies done outside our country*.

### What this study adds

- *Demographic and clinical profiles of patients presenting with this to identify at risk groups;*
- *Specifies outcome in terms of mortality in our setting and in our country contributed to by this group of patients;*
- *Provides comparative data to suggest direction for further research on factors associated with higher mortality in our setting*.
